# Human Disturbance and Geometric Constraints Drive Small Mammal Diversity and Community Structure along an Elevational Gradient in Eastern China

**DOI:** 10.3390/ani12151915

**Published:** 2022-07-27

**Authors:** Xiaoxin Pei, Xueyang Ren, Jiangxiao Hu, Kenneth Otieno Onditi, Yifan Xu, Min Zhang, Wenqing Chang, Zhongzheng Chen

**Affiliations:** 1 Collaborative Innovation Center of Recovery and Reconstruction of Degraded Ecosystem in Wanjiang Basin Co-Founded by Anhui Province and Ministry of Education, School of Ecology and Environment, Anhui Normal University, Wuhu 241002, China; peixiaoxin725@126.com (X.P.); renxueyang077@163.com (X.R.); jiangxiao202103@163.com (J.H.); iven0421@163.com (Y.X.); zm1763238579@163.com (M.Z.); 13225466718@163.com (W.C.); 2 State Key Laboratory of Genetic Resources and Evolution, Kunming Institute of Zoology, Chinese Academy of Sciences, Kunming 650201, China; kenotieno@hotmail.com; 3 Kunming College of Life Science, University of Chinese Academy of Sciences, Kunming 650201, China

**Keywords:** eastern China, elevational pattern, community structure, functional diversity, phylogenetic diversity, human disturbance

## Abstract

**Simple Summary:**

Biodiversity patterns and mechanisms along elevational gradients have long been the focus of conservation research. However, few studies have been conducted in mountainous areas of eastern China, especially for small mammals. In this study, we used a standard sampling method to survey small mammals along the gradient of Qingliang Mountain in eastern China and analyzed the patterns and mechanisms of diversity and community structure. We found inconsistencies between different diversity dimensions. Functional and phylogenetic structures were mainly clustered but showed opposite elevation patterns. Human disturbance and MDE were the main drivers of the diversity patterns, but with contrasting effects on different dimensions. These findings emphasize the importance of a multiple dimensions approach to biodiversity conservation and call for increased conservation efforts in the low and middle elevation regions.

**Abstract:**

Understanding the mechanisms influencing patterns and processes of biological diversity is critical to protecting biodiversity, particularly in species-rich ecosystems such as mountains. Even so, there is limited knowledge of biodiversity patterns and processes in the mountains of eastern China, especially about small mammals. In this study, we examined the taxonomic, functional, and phylogenetic diversity of small mammal distribution and community structure along the elevational gradient of Qingliang Mountain, eastern China. We then evaluated how they are influenced by space (area and mid-domain effect (MDE)), environment (temperature, precipitation, and normalized difference vegetation index (NDVI)), and human disturbance. The results showed hump-shaped patterns of taxonomic and phylogenetic diversity along elevation gradients, peaking at 1000 m, unlike functional diversity, which peaked at lower elevations (600 m). The mean pairwise distance and mean nearest taxon distance of functional and phylogenetic variance (MFD and MPD, respectively) were also incongruent. The MFD and MPD showed hump-shaped patterns along elevations; however, unlike MFD, which peaked at lower elevations (600 m), MPD peaked at higher elevations (1200 m). The mean nearest functional taxon distance (MNFD) decreased, while the mean nearest phylogenetic taxon distance (MNTD) increased along the elevation gradient. The higher elevations were functionally more clustered, while the lower elevations were phylogenetically more clustered, suggesting that environmental filtering for traits was stronger at higher elevations. In comparison, phylogenetic conservatism of ecological niches had a stronger influence at lower elevations. The diversity and community structure indices were inconsistently explained, with human disturbance and MDE accounting for the biggest proportions of the model-explained variances. Overall, the results confirm that environmental filtering and human disturbance significantly influence small mammals’ diversity and community structure. These findings also emphasize the need for increased conservation efforts in the middle and lower elevation regions of Qingliang Mountain.

## 1. Introduction

Mountains are home to nearly 87 percent of the world’s mammals, birds, and amphibians, despite only covering 25 percent of the total land area [1]. Due to notable environmental differences between elevation gradients, mountains portray unique vertical biodiversity patterns [2], making them ideal systems for researching biodiversity patterns and mechanisms and conservation [3,4]. Despite the high biodiversity they host, mountains experience high human activity pressure and climate change, and more studies are needed to enhance the efficacy of conservation measures [5,6]. Identifying the key biodiversity patterns and the corresponding drivers in mountain ecosystems is crucial for biodiversity conservation.

In the past few decades, mountain biodiversity patterns have attracted much interest, with most studies focusing on species richness [7,8,9,10,11]. These studies have highlighted four main elevational patterns in species richness: decreasing, low plateau, a low plateau with a mid-elevation peak, and a hump-shaped pattern [5]. Of these patterns, the hump-shaped pattern has emerged as the most common (45% of all cases), especially in non-flying small mammals (nearly 90% of all cases) [5]. These studies have also highlighted spatial, climatic, and habitat productivity variables as the most important drivers of mountain biodiversity patterns [4,9,12,13].

The mid-domain effect (MDE), which assumes spatial boundaries limit species dispersal and drive them towards the center of an area, resulting in a mid-domain peak in species richness [14], has been one of the most researched drivers of biodiversity patterns along elevation gradients. Like MDE, productivity is recurrently observed as a vital driver of species richness patterns, with positive relationships between productivity and diversity as communities with high productivity can support more individuals and thus more species [15,16]. Similarly, temperature and precipitation significantly affect species richness directly or indirectly [17,18,19,20], although they do not predict a single pattern along the elevation gradient, making it unclear how well they correspond to species richness [5]. Notably, human disturbance has increasingly emerged as an important driver of biodiversity patterns, with recent studies noting a strong negative effect of disturbance on diversity [21,22,23].

While previous studies of mountain biodiversity patterns offered an essential theoretical basis for diversity research and conservation efforts, most used species richness only and ignored or downplayed the influences of differences in species’ ecological functions and evolutionary history [24,25]. Thus, most previous studies may be over-simplistic or misleading for a holistic understanding of species assembling dynamics. Therefore, researchers have gradually opted for multidimensional community characterization approaches, incorporating species’ traits and phylogenetic differences into diversity measures to better reflect ecological and evolutionary processes [26]. Although some studies have found that elevational patterns of functional and phylogenetic diversity are congruent with species richness patterns [25], others have found them incongruent [24,27], suggesting that different mechanisms drive different biodiversity dimensions. Thus, a multidimensional diversity analysis accounting for taxonomic, functional, and phylogenetic diversity is essential to better understanding biodiversity patterns along elevational gradients.

Compared with studies on the elevational patterns of species richness and diversity, the community structure and the underlying drivers have been less studied. Studies have shown that community structure is mainly driven by environmental filtering, dispersal limitation [28], and biological interactions [29]. Environmental filtering constrains species within specific environmental conditions, eliminating those that cannot adapt to the limits [27,30,31]. As a result, species may undergo trait convergence to enhance their survival in particular habitats, such as high altitudes and deserts [32,33]. On the other hand, biological interactions often favor trait differentiation to minimize high competition pressure between closely related species [31,34]; it is the dominant driver of assembling dynamics in species-rich communities. Because co-existence mechanisms across environmental gradients [26] can lead to communities structured by multiple factors, it is essential to understand the role of these mechanisms in community assembly, as conclusions often differ between regions due to historical, climatic, and species composition variations [6].

Currently, research on elevational biodiversity patterns in China is mainly concentrated in the southwest regions, such as the Tibetan Plateau and Hengduan Mountains, with little research in eastern China, especially on small mammals. Qingliang Mountain, the highest mountain in the Tianmu Mountain Range, is not only a representative area of the hilly landscape in eastern China but also one of the 35 priority areas for biodiversity conservation in China. It has a complex topography that maintains a high level of biodiversity, including rare and endemic species [35,36]. The mountain’s small mammal diversity and community structure remain poorly understood. In the present study, we investigated the composition and elevational patterns of small mammal taxonomic, functional, and phylogenetic diversity and community structure in Qingliang Mountain for the first time. We trapped small mammals using standardized techniques and assessed the role of spatial (mid-domain effect (MDE)), environmental (mean annual temperature, annual precipitation, normalized difference vegetation index (NDVI)) variations, and human disturbance in determining the elevational patterns. Our aims were to (1) examine the elevational patterns of small mammals in Qingliang Mountain based on taxonomic, functional, and phylogenetic diversity, (2) analyze the functional and phylogenetic structures of small mammals along elevational gradients, and (3) assess the influence of environmental, spatial, and human disturbance factors on the diversity and community structure patterns.

## 2. Materials and Methods

### 2.1. Study Area

**Figure 1:**
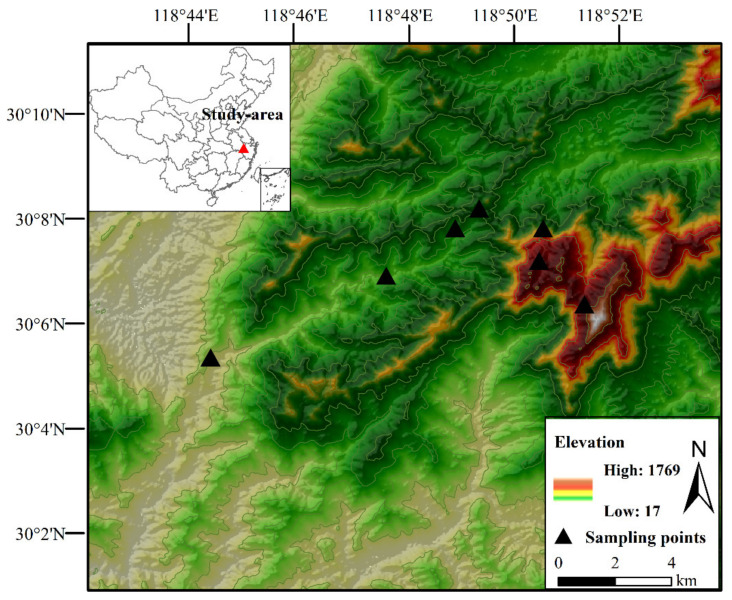
The map of the study area showing the extent of Qingliang Mountain, Anhui Province, China, and the sampling points.

This study was conducted in Qingliang Mountain, 30°03′–30°09′ N; 118°45′–118°53′ E (Figure 1), the main peak of Tianmu Mountain (elevation 1787 m) and the second-highest peak in eastern China. The vegetation of the mountain is stratified into zones along the elevation gradient, comprising evergreen broad-leaved forest (elevation < 700 m), broad-leaved mixed forest (700–1200 m), deciduous broad-leaved forest (1200–1500 m), and montane coppice, scrubs, meadows, and swamps (>1500). The National Nature Reserve, established in 2007, covers only the higher elevations (mostly > 1300 m), implying that the many farms and villages in the lower elevations may threaten the mountain’s biodiversity.

### 2.2. Sampling

From November to December 2019, we used standardized techniques to sample small mammals in Qingliang Mountain. Transects were laid along elevational gradients at intervals of 200 m, and trap lines were set at 25 m below or above the transect lines. Field surveys were conducted from 300 m (forest edge) to 1700 m (the top of the mountain). Consequently, seven transects were laid: at 400 (300–500) m; 600 (500–700) m; 800 (700–900) m; 1000 (900–1100) m; 1200 (1100–1300) m; 1400 (1300–1500) m; and 1600 (1500–1760) m. Four trap lines were set in key habitat types at each elevation band. The trap lines were set along the middle of each elevation band to reduce edge effects. Sherman traps were then placed at 10 m intervals along the trap lines. In addition, five plastic bucket pitfalls were placed at spots where shrews were likely to inhabit along the trap lines. Oats were used as bait in the Sherman traps, while no bait was used in the bucket pitfalls. The traplines were sampled for two consecutive nights, with traps checked once and rebaited the next morning.

In total, around 1000 trap nights were accumulated for each elevation band. We identified the captured individuals to the species level and recorded their body mass and external morphological trait measurements. Stuffed skins and cleaned intact skulls of select samples were also prepared as voucher specimens. All specimens collected for this study are stored at the Anhui Normal University, Anhui, China.

### 2.3. Species Trait Data

Thirteen traits, including five external morphological traits (body mass, head-body length, hind foot length, tail length, and ear length) and eight dental traits (upper incisors width, upper incisors depth, lower incisors width, rostrum length, rostrum width, upper cheek teeth row length, upper cheek teeth row width, jaw lever length) were selected for assessing functional diversity. These traits were suitable for inferring functional diversity as they represent species’ feeding and motor adaptations [37,38]. We used the ‘*K* statistic’ [39] to further select traits. The statistic quantifies phylogenetic signal (the propensity for related species to be functionally more similar to each other than to randomly drawn species within a community). Values of *K* ≥ 1 indicate the presence of a phylogenetic signal, with *K* close to 0 indicating a weak phylogenetic signal [39]. The ‘*K* statistic’ was calculated using the ‘multiphylosignal’ function in the R package *picante* [40]. Eventually, only traits with significant phylogenetic signals (*p* < 0.05) were retained (Table S1) for estimating functional diversity. The morphological and craniodental traits of specimens used in this study are shown in Table S2.

### 2.4. Phylogenetic Analyses

We used two mitochondrial genes (Cytochrome b, *CYTB*, and Cytochrome c oxidase subunit 1, *COI*) to infer an input phylogenetic tree for estimating phylogenetic diversity indices. The total genomic DNA of specimens was extracted using a DNA extraction kit (Qiagen DNeasy Blood and Tissue Kit, Beijing, China). The complete *CYTB* and *COI* genes were amplified using primers and PCR conditions from He et al. [41]. The PCR products were purified and sequenced in both directions using the BigDye Terminator Cycle kit v.3.1 (Invitrogen, Waltham, MA, USA) on an ABI 3730xl sequencer (Applied Biosystems, Waltham, MA, USA).

The resulting sequences were edited using DNASTAR Lasergene SeqMan 7.1 and aligned with MEGA-X. We then used Bayesian inference (BI) to reconstruct the phylogenetic relationships of specimens in PhyloSuite [42] based on the best-fit partitioning schemes estimated using PartitionFinder v.2.0. The posterior distributions were calculated by Markov chain Monte Carlo (MCMC) sampling from 20 million generations. The first 25% of the samples were discarded as burn-in before the trees were summarized into a single tree (based on Maximum clade credibility) using TreeAnnotator v2.6.4. The tree was then viewed, labelled, and exported in FigTree 1.4.4. It was used as input for estimating the phylogenetic diversity and structure indices (Figure S1).

### 2.5. Diversity Indices

We calculated small mammals’ taxonomic, functional, and phylogenetic diversity in each elevation band. We used the observed species richness to measure taxonomic diversity, Rao’s quadratic entropy (RaoQ) to measure functional diversity, and Faith’s phylogenetic diversity index to measure phylogenetic diversity [43]. The RaoQ index is calculated as the sum of weighted trait differences and represents the mean distance of traits between two random individuals [44]. Before estimating RaoQ, we first used Gower distance to calculate pairwise functional dissimilarity distances between all species. RaoQ was calculated using the function ‘dbFD’ in R package *FD* [45]. Phylogenetic diversity was calculated using the function ‘pd’ in the R package *picante*. We also calculated the mean pairwise phylogenetic distance (MPD) and the mean pairwise functional distance (MFD) to evaluate the overall phylogenetic and functional dissimilarity in communities. Because MFD and MPD cannot detect finer-scale phylogenetic patterns, we further calculated the mean nearest functional taxon distance (MNFD) and the mean nearest phylogenetic taxon distance (MNTD) to assess the average phylogenetic and functional distance of each species to its closest relative in communities [46]. The MPD and MFD were calculated using the R package *picante* with the ‘mpd’ function. The MNTD and MNFD were calculated with the same package using the ‘mntd’ function.

### 2.6. Functional and Phylogenetic Structure

We calculated the nearest taxon index (NTI) and net relatedness index (NRI) based on the mean pairwise phylogenetic and functional taxon distances and mean nearest phylogenetic and functional taxon distances. These indices were used to infer community assembly processes when traits were conserved (with a significant phylogenetic signal). The NRI measures the relatedness between taxa at the base of the phylogenetic tree, reflecting the basal-weighted community assembly dynamics. The NTI measures the relatedness of the nearest taxa at the tip of a tree, reflecting tip-weighted community dynamics [47]. The NRI and NTI values < 0 (overdispersion) indicate that competitive exclusion may be the dominant driver of community structure. In contrast, NRI and NTI values > 0 (clustering) indicate that environmental filtering may be the dominant driver of community structure [41]. The NRI and NTI values > 1.96 reflect significant clustering, while NRI and NTI values < −1.96 reflect significant overdispersion [47]. The NTIs were calculated using the ‘ses.mntd’ function in R using the *picante* package while the NRIs were calculated using the ‘ses.mpd’ function in the same package.

### 2.7. Explanatory Variables

The 3D surface area of each 200 m elevational band from 300 to 1700 m was calculated in ArcGIS 10.6 (ESRI, Redlands, CA, USA) based on the GDEM 30-m digital elevational data (NASA, https://www2.jpl.nasa.gov/srtm/, accessed on 12 April 2016). The MDE values were estimated using RangeModel5 [48]. Annual precipitation and mean annual temperature features were extracted from WordClim (https://www.worldclim.org, accessed on 14 March 2020) at the 30s (1 km^2^) resolution. As a proxy for the total vegetation cover, the normalized difference vegetation index (NDVI) was obtained from NASA MOD13 products (https://ladsweb.modaps.eosdis.nasa.gov/search/order/3, accessed on 21 March 2021) for November and December 2019. Human disturbance (HD) was estimated as the proportion of artificial land coverage from the Globeland30 (http://www.globallandcover.com, accessed on 27 March 2021) [49] land cover data product. All data sampling was performed in ArcGIS 10.6 (ESRI, Redlands, CA, USA).

### 2.8. Data Analyses

We used polynomial regression to explore and plot the elevational distribution of different diversity dimensions. The bivariate correlation between diversity indices was calculated using Pearson Correlation Coefficients.

Hierarchical partitioning was used to estimate the explanatory power of the five variables on diversity variances. The analysis was performed using the R package *hier.part*. Hierarchical partitioning [50] can reduce the effect of variable collinearity and can be used to determine the proportion of diversity variances accounted for by predictor variables.

Further, we also conducted multiple regression analyses to explore the main explanatory variables driving diversity variances along the elevation gradient. Before the multiple regression analysis, all variables were z-score standardized, and their normality and homoscedasticity checked. We calculated the variance inflation factor (VIF) of each variable in the models to check for and handle multicollinearity, with only variables with VIFs < 10 consequently considered [4,24]. Automated model selection was performed in R using *MuMIn*, ranked by corrected Akaike information criterion (AICc). Model averaging with the ‘model.avg’ function in R package *MuMIn* was used to combine models with nearly-equivalently supported models (ΔAICc < 2).

## 3. Results

**Figure 2:**
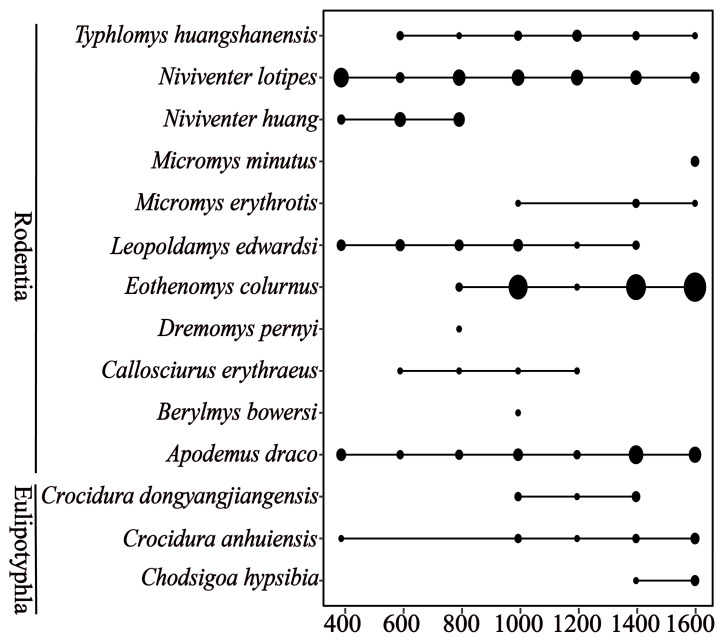
The elevational distribution ranges of small mammal species in Qingliang Mountain used in this study.

We trapped 508 individuals of small mammals, comprising 14 species in 10 genera from over 6500 trap nights, at a trap success rate of 7.92%. The number of individuals trapped per elevational band ranged from 28 to 128, and species ranged from five to 10. Among the 14 species, three belonged to the order Eulipotyphla and 11 were in the order Rodentia. The elevational range of each species is shown in Figure 2.

**Figure 3:**
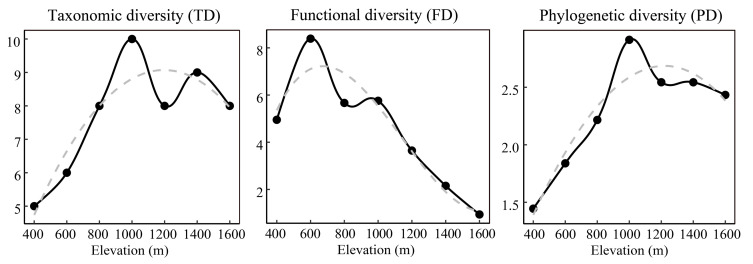
Elevational patterns of small mammal taxonomic diversity (TD), functional diversity (FD), phylogenetic diversity (PD) along a 1400 m elevational gradient in Qingliang Mountain, Anhui, China. The gray dashed line represents the best-fit line.

**Figure 4:**
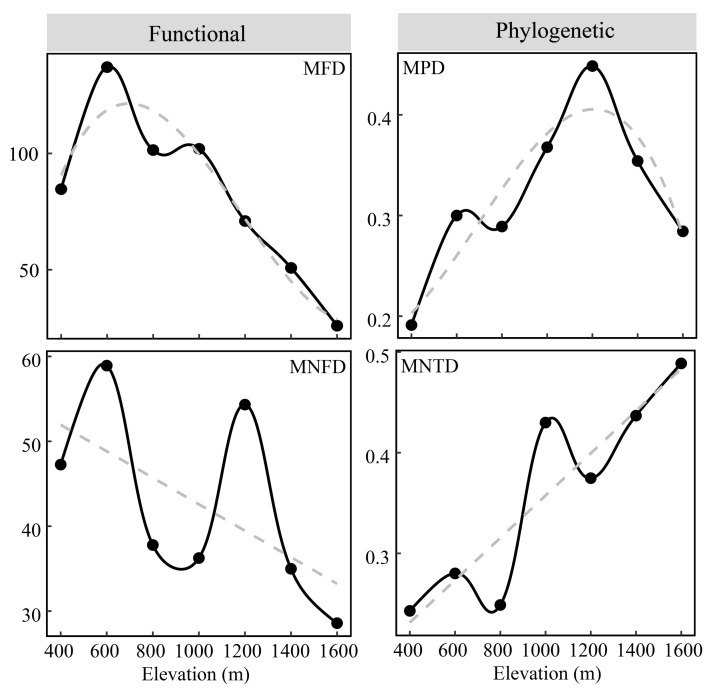
Elevational patterns mean pairwise functional distance (MFD), mean pairwise phylogenetic distance (MPD), mean nearest functional distance (MNFD), and mean nearest taxon distance (MNTD) of small mammals along a 1400 m elevation gradient in Qingliang Mountain, Anhui, China. The gray dashed line represents the best-fit line.

Taxonomic, functional, and phylogenetic diversity showed hump-shaped patterns with elevation, unlike functional diversity (Figure 3). Taxonomic and phylogenetic diversity peaked around 1000 m, while functional diversity peaked around 600 m. The MFD exhibited a pattern almost similar to functional diversity, peaking at 600 m, while the MPD showed a hump-shaped pattern, peaking at 1200 m. The MNFD decreased while MNTD linearly increased as elevation increased (Figure 4). Pearson correlation results showed that taxonomic and phylogenetic diversity were strongly correlated (but not significantly) with functional diversity; there were no significant correlations between other phylogenetic and functional diversity indices (Table 1).

**Figure 5:**
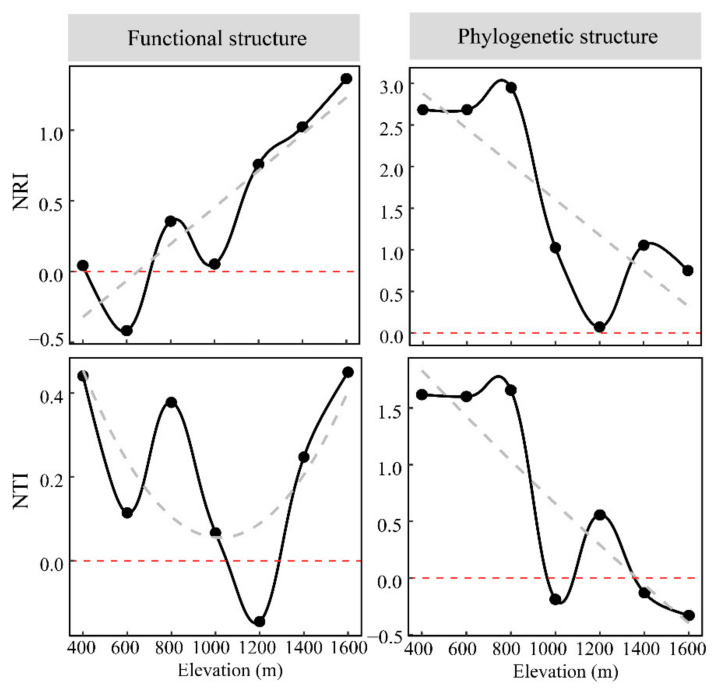
The patterns of phylogenetic and functional structure of small mammal communities along the elevational gradients in Qingliang Mountain. The gray dashed line represents the best-fit line.

Although NRI and NTI showed assemblages were mainly clustered, there were significant differences between indices at high and low elevations. The functional NRI values above 1000 m elevations were usually higher than below 1000 m, indicating that the functional structure was relatively more clustered at the higher elevations. The functional NRI was inconsistent with the NTI. The NTI did not significantly vary across elevation gradients. In contrast, the phylogenetic NRI and NTI were lower at elevations below 1000 m and above 1000 m, with NRI > 2 below 1000 m, indicating that lower elevations were phylogenetically more clustered (Figure 5).

**Figure 6:**
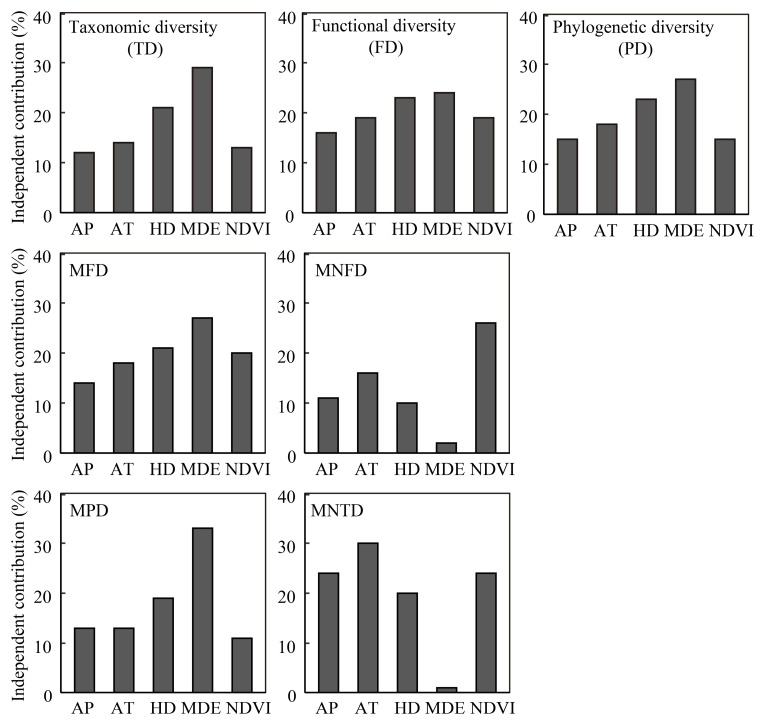
The proportion of the independent contribution of each predictor variable to model-explained diversity variances derived by hierarchical partitioning. Abbreviations: TD, taxonomic diversity; FD, functional diversity; PD, phylogenetic diversity; MFD, mean pairwise functional distance; MPD, mean pairwise phylogenetic distance; MNFD, mean nearest functional taxon distance; MNTD (P), mean nearest phylogenetic taxon distance; AP, annual precipitation; AT, mean annual temperature; HD, human disturbance; MDE, the mid-domain effect; NDVI, normalized difference vegetation index.

The hierarchical partitioning indicated that MDE and human disturbance were the most important factors for taxonomic, functional, and phylogenetic diversity, MFD, and MPD variances (Figure 6). Respectively, MDE respectively explained 29%, 24%, 27%, 27%, and 33% of the variances, while human disturbance explained 21%, 23%, 23%, 21%, and 19%. The MNFD was best explained by NDVI (26%) and mean annual temperature (16%), while MNTD was best explained by mean annual temperature (30%), annual precipitation (24%), and NDVI (24%).

Similar to hierarchical partitioning, model selection showed that MDE and human disturbance were the strongest explanatory variables for most diversity indexes (Table 2). The difference was that human disturbance became more important (with higher standardized beta coefficients) than MDE for taxonomic, functional, and phylogenetic diversity and MFD. Human disturbance was the only factor negatively correlated with taxonomic and phylogenetic diversity. Human disturbance and MDE were positively correlated with functional diversity and MFD, while MDE was the only factor retained in the best model for MPD. NDVI and mean annual temperature had the strongest contribution (negative correlation) to the MNFD and MNTD variance, respectively.

Model averaging for the top models with ΔAICc < 2 for functional diversity, MPD, MFD, and MNFD yielded similar results to model selection, with human disturbance and MDE being the two most important predictors of functional diversity and MFD variances. The MDE was also the most important factor for MPD, while NDVI was the most important explanatory factor for MNFD (Table S3).

## 4. Discussion

### 4.1. Elevational Patterns of Small Mammal Diversity

In the present study, the elevational patterns of small mammals in Qingliang Mountain were studied for the first time. We found that small mammal taxonomic diversity patterns along the elevational gradients were hump-shaped, peaking at about 1000 m (Figure 3). This pattern is consistent with most previous studies of small mammals in different regions [51,52] and appears to be the dominant pattern along elevational gradients. Functional and phylogenetic diversity also showed similar patterns along elevational gradients. However, while elevational diversity curves of phylogenetic and taxonomic diversity were similar, the functional diversity curve was significantly different. Specifically, the phylogenetic diversity peaked at 1000 m, like taxonomic diversity, while functional diversity peaked at lower elevations (600 m).

The correlation analyses showed that taxonomic diversity was highly correlated with phylogenetic biodiversity, unlike functional diversity, which was not significantly correlated with taxonomic or phylogenetic diversity (Table 1). These results concur with previous studies where incongruences between functional and phylogenetic diversity have been frequently reported [24,26,27], although other studies have reported consistencies [25]. The MFD and MNFD were higher at lower elevations compared to higher elevations, unlike MPD and MNTD, which portrayed greater functional variation at lower elevations and greater phylogenetic variation at higher elevations (Figure 4). In addition to inconsistencies between function and phylogeny, there were inconsistencies between MFD and MNFD and between MPD and MNTD, suggesting that trait and phylogenetic variation differed between basal-weighted and tip-weighted metrics. These inconsistencies provide additional insight into the changing phylogenetic and functional dynamics of biodiversity [53]. Overall, our results highlight the importance of multidimensional analysis of biodiversity and call for caution when using any single diversity component as a surrogate for others.

### 4.2. Community Assembly Mechanisms

Although the patterns of functional NRI and NTI along the elevation gradient were inconsistent, they were positive, except for NRI in the 600 m bands and NTI in the 1200 m bands, where values were <0 (Figure 5). In this study, we selected traits with significant phylogenetic signals to analyze functional diversity and structure; therefore, communities can be considered functionally and phylogenetically clustered when NRI or NTI values were >0 [24]. As such, the results suggest that the small mammal community in Qingling Mountain functionally clustered overall, with the basal-weighted clustering significantly greater at high altitudes than at low altitudes. Such a pattern suggests that resource limitation/environmental filtering at high altitudes may have led to high similarity in small animal traits [54]. The functional NTI was not significantly different across elevations, and traits were mainly conserved, indicating that homogeneity in functional structure is weak when considering the closest relatives, potentially reducing competition [55]. Since NTI measures the distance to the closest relative, it is a more robust index for detecting limiting similarity. Our results suggest that limiting similarity may not be the primary factor driving the observed functional clustering [56,57,58], especially at high elevations. Instead, trait convergence may be the main reason for the functional clustering in Qingliang Mountain. Such convergence of traits generally results from strong environmental filtering necessitating species exploiting similar niches to have trait plasticity, which may be independent of common phylogenetic ancestry [58,59].

The phylogenetic NRI was always positive and decreased with elevation, indicating clustered phylogenetic structure, with assemblages notably over-clustered at low elevations and the degree usually decreasing as elevation increased. This pattern suggests that co-occurring species are more closely related phylogenetically at lower elevations [27]. At low elevations, phylogenetic clustering may result from intensive human disturbance creating suitable habitats for some species and eliminating those that are intolerant of the disturbed environments [27,60,61]. In addition, the phylogenetic NTI and NRI had similar elevational patterns, with the degree of clustering typically decreasing with elevation. The NTI was >1 at lower elevations but usually <0 at higher elevations, indicating that the tip-weighted phylogenetic structure was strongly clustered at lower elevations and mainly dispersed at higher elevations. This pattern suggests that the dispersed phylogenetic structure when considering closest relatives may arise from competition for limited resources in stressful environments (cold, wet), typical at higher elevations [24]. Combining the results of phylogenetic NTI and NRI suggests that community dynamics between closest relatives dominate the overall phylogenetic structure. In contrast, limited phylogenetic similarity may drive the phylogenetic clustering of communities at lower elevations [57]. Notably, the restricted phylogenetic differences for the tip-weighted dimensions may lead to an unstable phylogenetic structure of the overall community at high altitudes.

Furthermore, the phylogenetic and functional structure patterns along the elevation gradient were incongruent, an observation frequently discussed in past studies [24,62]. This pattern suggests that trait and phylogenetic weights may not be fixedly associated [58], implying that relying solely on phylogenetic or functional dimensions to reveal community structure is inefficient. Combining the functional and phylogenetic effects is crucial to better understanding community assembly processes [24,59].

### 4.3. Drivers of Diversity Patterns

Our results support the idea that small mammal diversity along elevation gradients is affected by multiple factors, with human disturbance and MDE being the most significant factors in Qingliang Mountain. Both model selection and hierarchical partition demonstrated strong associations between human disturbance and MDE and diversity dimensions. The MDE emphasizes the role of geometric boundaries in constraining species ranges, leading to a mid-elevation peak of species richness [63]. It has frequently been supported as a key determiner of elevational patterns of species richness [51,60,64]. The predictive power of MDE is strongly affected by the boundaries of the domain, which requires studies to cover the “hard” boundaries for organisms [14]. The study area ranged in elevation from 300 m (forest edge) to 1700 m (mountain peak). Both elevation limits are potentially rigid boundaries for some small mammals, which might explain the high explanatory power of MDE in our study and highlight the importance of studying the whole elevation gradient when evaluating the fit of MDE.

The MDE hypothesis was initially proposed to address taxonomic diversity patterns [14]. However, because the geographic traits such as geographic range size or phenotypic characteristics are tied to particular species, the MDE might create gradients in other biodiversity dimensions [65]. In our study, the hierarchical partitioning indicated that MDE was the most important factor for species richness, taxonomic, functional, and phylogenetic diversity, and MFD and MPD (Figure 6). The MDE was also retained in the best models of functional diversity, MFD, and MPD, indicating it substantially influences the functional and phylogenetic diversity of small mammals in Qingliang Mountain. Still, more research is needed on how and to what extent MDE affects functional and phylogenetic biodiversity.

Studies have reiterated that human disturbance negatively affects multiple aspects of biodiversity across regions and taxa [27,61,66,67]. Human activities often lead to habitat fragmentation, degradation, and loss [68], resulting in widespread disruption of animal movement, diel activity, reproduction, and survival [66]. Farms, villages, and roads are often widespread at low elevations, negatively impacting biodiversity by eliminating species incapable of adapting to the disturbed environments [27]. In Qingliang Mountain, human disturbance had a clear negative correlation with the taxonomic and phylogenetic diversity, suggesting that the home ranges of some species gradually decreased as human disturbance increased [67,69]. This was indirectly supported by the NRI and NTI of phylogenetic structure at lower elevations. Unexpectedly, human disturbance had a positive explanatory influence on functional diversity and MFD, which is inconsistent with previous studies where human disturbance negatively affected functional diversity [27,66,70]. Notably, however, positive effects of human disturbance on birds’ functional diversity have been recorded in the Himalayas [24,71], and on certain plants in the Yak Meadow Park, Mount Jade Dragon National Geological Park of Yunnan, China [72]. The positive effects of human disturbance on small mammal functional diversity in our study may have resulted from human activities creating suitable habitats for some species with unique traits (e.g., big-size species), such as *Niviventer huang* and *Leopoldamys edwardsi*.

### 4.4. Conservation Implications

For a long time, conservation strategies focused on species richness and ignored the importance of phylogenetic and functional diversity in the ecosystem. Here, we observed incongruences between taxonomic, functional, and phylogenetic diversity dimensions along elevation gradients, providing complementary insights into community structure and diversity. A multidimensional analysis of biodiversity is urgently needed to enhance the efficacy of biodiversity conservation and ecosystem management. In the study area, the nature reserves only cover the higher elevations (>1300 m). However, we found that the taxonomic and phylogenetic diversity peaked at 1000 m, while the functional diversity peaked at 600 m (Figure 3). Therefore, the current protected areas are insufficient for conservation; conservation efforts should be increased in the middle and lower elevations.

Also, our study revealed that human disturbance is a major factor shaping small mammal diversity. In Qingliang Mountain, many villages and farms are found below 1000 m, which also receives many tourists yearly. Human activity appears to strongly impact local small mammal biodiversity by changing the community structure. Such human influence on biodiversity patterns is likely common in other mountains in eastern China. The protected areas should be expanded to control the negative impacts of human activities in key biodiversity areas, such as Qingliang Mountain.

## 5. Conclusions

We studied the elevation pattern of small mammals in Qingliang Mountain for the first time. We found that taxonomic, functional, and phylogenetic diversity portray a hump-shaped pattern in general, but functional diversity peaked at a much lower elevation than taxonomic and phylogenetic diversity. We also found spatial incongruence between MPD, MFD, MNFD, and MNTD. The incongruences between diversity dimensions suggest that multidimensionality is critical for understanding species diversity and community structure patterns. Furthermore, we found that human disturbance and MDE were the primary drivers of small mammal diversity and community structure along the elevation gradient in Qingliang Mountain. Overall, our results call for caution in using any single diversity component as a surrogate for others and increased conservation efforts in the middle and lower elevations of Qingliang Mountain.

## Figures and Tables

**Table 1:** Bivariate Pearson correlation coefficients between multiple diversity dimensions of small mammals in Qingliang Mountain.

Index	SR	FD	PD	MPD	MFD	MNTD	MNFD
SR	1 ***						
FD	−0.341	1 ***					
PD	0.973 ***	−0.372	1 ***				
MPD	0.672	−0.154	0.778 *	1 ***			
MFD	−0.293	0.996 ***	−0.321	−0.089	1 ***		
MNTD	0.707	−0.73	0.771 *	0.482	−0.694	1 ***	
MNFD	−0.588	0.635	−0.482	0.123	0.648	−0.595	1 ***

SR, Species richness; FD, Functional diversity; PD, Phylogenetic diversity; MFD, the mean pairwise functional distances; MPD, the mean pairwise phylogenetic distance; MNFD, the mean nearest functional distance; MNTD, the mean nearest taxon distance. * *p* < 0.05; *** *p* < 0.001.

**Table 2:** Results of best model selection exploring associations between multiple biodiversity indices and five predictor variables along elevation gradients in Qingliang Mountain.

Multidimensional Metrics	Standard Coefficient of the Best Model						
	MDE	HD	NDVI	AT	AP	R^2^_adj_	AICc
Species richness (SR)		−0.796				0.561	25.754
Functional diversity (FD)	0.600	0.914				0.948	23.280
Phylogenetic diversity (PD)		−0.846				0.659	23.982
MFD	0.644	0.886				0.941	24.112
MPD	0.726					0.432	27.545
MNFD			−0.727			0.434	27.531
MNTD				−0.904		0.780	20.926

MFD, the mean pairwise functional distances; MPD, the mean pairwise phylogenetic distance; MNFD, the mean nearest functional distance; MNTD, the mean nearest taxon distance; MDE, the mid-domain effect; AT, mean annual temperature; AP, annual precipitation; NDVI, normalized difference vegetation index; HD, human disturbance. R^2^_adj_ is the adjusted r^2^ value for multiple regressions.

## Data Availability

The datasets generated during and/or analysed during the current study are not publicly available as it is currently being used for other research but are available from the corresponding author on reasonable request.

## Supplementary Materials

The following supporting information can be downloaded at: https://www.mdpi.com/article/10.3390/ani12151915/s1, Figure S1: Bayesian tree based on COI and cytb genes of 14 small mammalsin Qingliang Mountain, eastern China.; Table S1: Phylogenetic signals of thirteen functional traits; Table S2: List of thirteen functional traits associated with morphology; food acquisition and exercise.
