# Supplementary material for: Human Disturbance and Geometric Constraints Drive Small Mammal Diversity and Community Structure along an Elevational Gradient in Eastern China
Source: Animals (Basel). 2022 Jul 27;12(15):1915. doi: 10.3390/ani12151915 (PMC9367490; doi:10.3390/ani12151915)
Supplement: Supplementary file 1 [file animals-12-01915-s001.zip › animals-1806065-supplementary.pdf]

Supplementary Figure and Tables

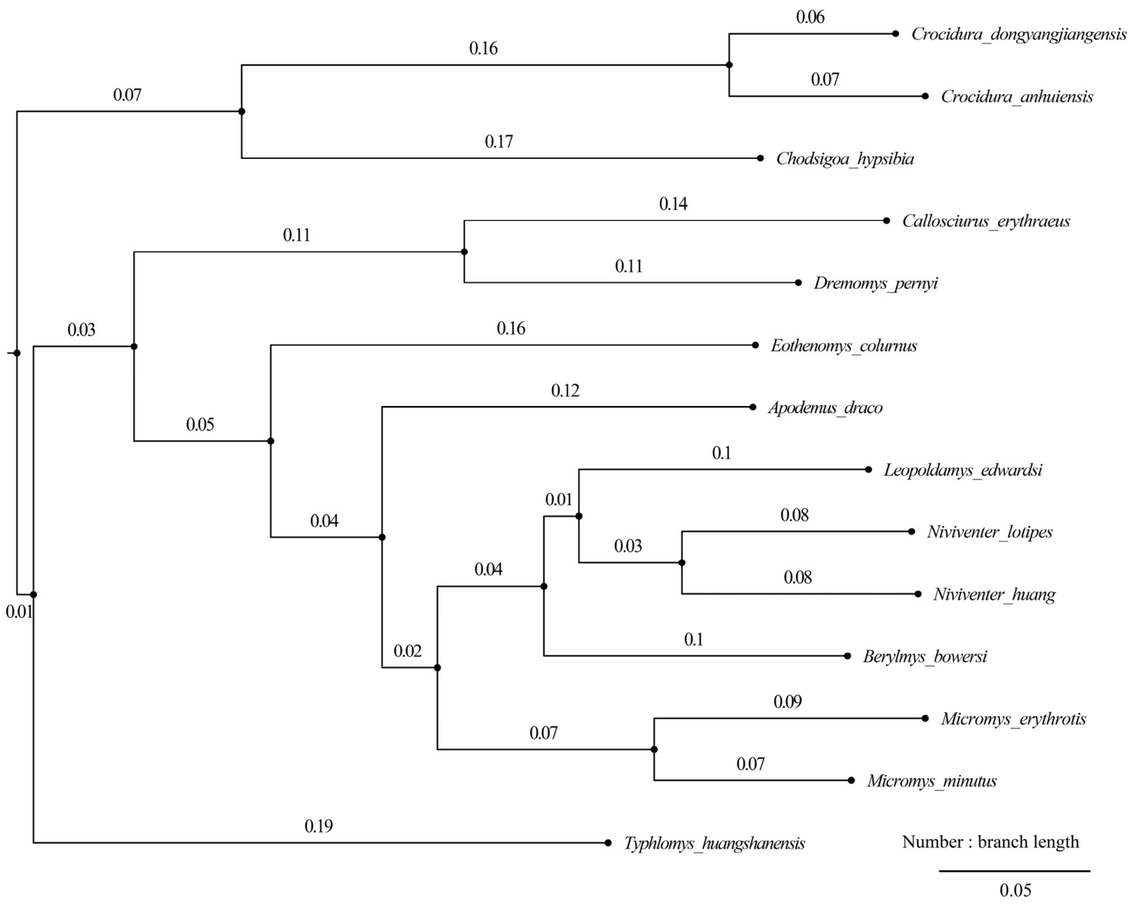

Figure S1. Bayesian tree based on COI and cytb genes of 14 small mammalsin Qingliang Mountain, eastern China.

Table S1. Phylogenetic signals of thirteen functional traits.

Traits	K value	P
Upper incisor width	0.78	0.05
**Upper incisor depth**	**0.98**	**0.01**
Lower incisor width	0.77	0.06
Upper cheek teeth row length	0.79	0.05
**Rostrum length**	**0.82**	**0.03**
**Rostrum width**	**0.90**	**0.02**
**Jaw-lever length**	**0.88**	**0.03**
**Upper cheek teeth row width**	**0.89**	**0.03**
Weight	0.61	0.21
Head and body length	0.77	0.06
**Tail length**	**0.90**	**0.03**
**Hind foot length**	**0.85**	**0.04**
**Ear length**	**0.88**	**0.04**

Traits in boldface were used to measure functional diversity

Table S2. List of thirteen functional traits associated with morphology; food acquisition and exercise.

Species	W	HBL	TL	HFL	EL	UIW	UID	LIW	UCL	RL	RW	JFL	UCW
Apodemus draco	17.74	84.28	90.02	20.69	15.12	1.96	1.22	1.48	3.80	6.29	4.50	9.82	4.94
Berylmys bowersi	350.10	228.00	248.00	53.00	30.00	4.83	2.76	3.91	8.81	16.00	8.68	22.35	10.51
Callosciurus erythraeus	160.00	179.00	153.50	46.00	21.00	3.54	2.27	2.71	8.63	13.57	8.18	21.40	12.45
Chodsigoa hypsibia	5.42	66.20	56.00	13.20	8.40	1.19	0.49	1.06	3.29	5.00	2.40	6.68	5.10
Crocidura anhuiensis	9.68	77.00	58.50	12.25	10.50	1.90	0.62	1.25	4.26	6.76	3.20	8.41	6.29

Crocidura attenuata	9.46	74.89	55.89	13.78	10.00	1.64	0.54	1.26	4.40	6.74	3.28	8.25	6.38
Crocidura dongyangjiangensis	4.69	60.67	45.17	11.25	8.50	1.17	0.42	0.94	3.53	5.24	2.78	6.57	5.04
Dremomys pernyi	155.06	180.00	100.00	40.00	16.00	4.23	2.51	2.84	8.37	16.26	8.64	20.79	11.13
Eothenomys colurnus	20.06	99.04	38.30	16.10	10.68	2.87	1.40	2.15	5.85	7.30	5.16	11.56	5.20
Leopoldamys edwardsi	334.45	231.69	272.96	49.96	34.31	4.79	3.06	3.80	10.18	16.70	9.93	23.08	11.68
Micromys erythrotis	6.54	55.75	64.25	14.00	9.63	1.51	0.91	0.86	2.84	4.21	3.02	6.77	3.53
Micromys minutus	6.80	55.00	66.50	14.88	10.00	1.45	0.82	1.11	3.28	4.79	2.98	6.92	4.45
Niviventer huang	44.61	116.38	149.77	26.19	19.12	2.66	1.77	2.07	5.43	9.28	5.60	12.75	6.58
Niviventer lotipes	38.13	111.83	147.22	25.38	20.04	2.50	1.65	1.94	5.43	8.92	5.53	12.33	6.56
Typhlomys huangshanensis	14.03	75.31	102.15	19.38	14.29	2.05	1.26	1.32	3.65	6.04	4.75	8.63	5.22

UIW: Upper incisor width; UID: Upper incisor depth; LIW: Lower incisor width; UCL: Upper cheek teeth row length; RL: Rostrum length; RW: Rostrum width; JFL: Jaw-lever length; UCW: Upper cheek teeth row width; W: Weight; HBL: Head and body length; TL: Tail length; HFL: Hind foot length; EL: Ear length.

Table S3. Model-averaging estimate, unconditional standard errors (SE), and 95% confidence interval (CI) of variables for multidimensional metrics.

Metrics	factor	Estimate	$SE_{.adj}$	z value	*P*	95% IC
Functional diversity (FD)	Human disturbance	0.43	0.47	0.92	0.36	0.47-1.30
	MDE	0.24	0.31	0.77	0.44	0.29-0.90
	NDVI	-0.15	0.35	0.44	0.66	-1.48- -0.17
	Annual temperature	0.12	0.32	0.38	0.70	0.13-1.50
	Annual precipitation	-0.05	0.21	0.23	0.82	-1.54-0.05
MPD	MDE	0.16	0.35	0.45	0.65	-0.06-1.51
	Human disturbance	-0.08	0.27	0.31	0.76	-1.55-0.21
	Annual precipitation	0.05	0.21	0.23	0.82	-0.31-1.53
	Annual temperature	-0.03	0.17	0.17	0.87	-1.57-0.5
	NDVI	0.01	0.12	0.08	0.94	-0.95-1.59
MFD	Human disturbance	0.36	0.45	0.81	0.42	0.42-1.30
	MDE	0.23	0.32	0.71	0.48	0.29-0.98
	NDVI	-0.19	0.38	0.49	0.63	-1.49- -0.15
	Annual temperature	0.11	0.30	0.35	0.72	0.06-1.52
	Annual precipitation	-0.04	0.19	0.21	0.84	-1.56-0.14
MNFD	NDVI	-0.17	0.37	0.46	0.64	-1.55-0.09
	Annual temperature	0.05	0.23	0.22	0.82	-0.44-1.64
	Human disturbance	0.04	0.19	0.20	0.84	-0.40-1.54
	Annual precipitation	-0.02	0.15	0.12	0.90	-1.61-0.72
	MDE	0.00	0.08	0.04	0.97	-0.95-1.31

MFD, the mean pairwise functional distances; MPD, the mean pairwise phylogenetic distance; MNFD, the mean nearest functional distance; MNTD, the mean nearest taxon distance; MDE, the mid-domain effect; NDVI, normalized difference vegetation index. R^2_{adj} is the adjusted r^2 value for multiple regressions.
